# Hospital Deputation to the Chancellor of the Exchequer

**Published:** 1911-07-29

**Authors:** 


### HOSPITAL DEPUTATION TO THE CHANCELLOR OF THE EXCHEQUER.

In his private room at the House of Commons on Tuesday, July 25, the Chancellor of the Exchequer received a deputation, which represented to him the effect which the National Insurance Bill might have upon the work of the hospitals of the country. Mr. Lloyd George was accompanied by the First Lord of the Admiralty (Mr. McKenna), the President of the Local Government Board (Mr. Burns), and the Attorney-General (Sir Rufus Isaacs).

The deputation consisted of the following:—*Representing the British Hospitals Association*: Sir William Cobbett, chairman Manchester Royal Infirmary; Mr. Chas. Lupton, treasurer Leeds General Infirmary; Dr. D. J. Mackintosh, M.V.O., medical superintendent Glasgow Western Infirmary; Mr. Howard Collins, house-governor Birmingham General Hospital; Mr. Conrad W. Thies, secretary Royal Free Hospital; and Mr. A. William West, treasurer St. George's Hospital, joint hon. secretary of the British Hospitals Association. *Representing the Central Hospital Council for London*: Mr. A. William West, treasurer St. George's Hospital; Mr. S. M. Quennell, secretary Westminster Hospital. *Representing Hospital Saturday Fund*: Sir Savile Crossley, Bart., and Mr. A. W. Davis, secretary Hospital Saturday Fund. The Hon. Sydney Holland also attended.

### The Views of the Deputation.

The deputation was introduced by Sir A. Haworth, who said that the British Hospitals Association recently held a meeting which was attended by the representatives of two hundred hospitals in the country. It was then felt that those voluntary hospitals were likely to be jeopardised very largely in their work by the Insurance Bill. That deputation was solely interested in the subject from a philanthropic point of view.

Sir William Cobbett, Chairman of the Manchester Royal Infirmary, said that for the time being he had the honour to represent the provincial hospitals of the United Kingdom. Meetings had been held in London, Manchester, Birmingham, and other centres, and they had had communications from numerous hospitals throughout the country supporting their view that the Bill, as it now stood, would seriously injure the voluntary hospitals financially. The case of the London hospitals perhaps differed to some extent from the provincial hospitals. Their resources were derived from other sources to a more considerable extent than those of the provincial hospitals. It might be that it might not affect them to the same extent that it would affect the provincial hospitals. The two most important classes of contributors to the provincial hospitals were the working-men and their employers. It was the universal opinion of the administrators of hospitals in the provinces that the effect of the Bill would be that the contributions of the workmen would cease and that the contributions of the employers would be very largely reduced. It was the universal opinion of those who had for years past been engaged in collecting and obtaining money for the purpose of carrying on those hospitals, that it must have the effect of closing some hospitals and very severely crippling others. He quoted the case of a hospital at St. Helens, with two hundred beds, where nobody was admitted as a patient unless he subscribed not less than a penny a week. The payments from that source amounted to £4,200 per annum, and only £139 was received from people who were not subscribers. If the workpeople's pence ceased, the hospital must close.

### The Case of the Chesterfield Hospital.

Then there was the case of the Chesterfield and North Derbyshire Hospital. Last year the total income was £6,000, of which £3,400 came from the workmen and £800 from the employers. In that case the result would be the same. He admitted that the word surmise might be applied to their anticipations, but at any rate the whole body of skilled administrators of hospitals were of the same opinion. At the Manchester Royal Infirmary they were helped by the contributions of the employers more than by the men, by legacies and donations. They had been endeavouring for some years past to increase their annual subscriptions. Their voluntary contributions amounted to about thirteen thousand pounds a year, and largely came from small employers. They anticipated that if the Bill passed in its present form those contributions would largely, if not wholly, cease. They had been employing special canvassers to increase their annual subscriptions, who, until the Bill was introduced, had been bringing in £18 a week apiece. They now reported that the employers would wait until they saw whether the Bill went through in its present form. If it did they would never give them anything.

Mr. Lloyd George: And you really believe that the rich employers of the neighbourhood will feel under no obligation to the hospital because they are paying the half contribution?

Sir William Cobbett stated that he was thinking more of the smaller employer, who made up a large part of the hospital's income. Large and rich firms would not like at first, at any rate, to withdraw their subscriptions. He did not suppose that they would at first, but he thought that it would be the beginning of the end with them. The small man would do it at once, and they would lose a large part of their £13,000. There was an unanimous body of opinion which thought that. Their positions as voluntary hospitals ought to be continued, because there was a vast amount of good work done throughout the country without remuneration. The remedy which they proposed was shortly this. They thought that the treatment of insured persons in the hospitals ought to be made a benefit and placed on the same footing, approximately, as the treatment of consumptives in sanatoria. Health committees ought to be directed to make arrangements with regard to the hospitals. They did not tie themselves down to that, for some better way might be found. They were not there to say that that was the only way.

### The Doctors' Complaint.

Mr. Lloyd George : It is one of the complaints of the medical profession that if you contribute towards the hospitals, you are contributing towards institutions which are competing with the medical profession. That is the view of the doctors.

Sir William Cobbett : Does that apply to the in- or out-patients, because nobody uses the hospitals for in-patients more than the doctors themselves?

Mr. Lloyd George : I think it is the out-patients.

Sir William Cobbett : My experience of club doctors is that as soon as a club doctor has had enough of the out-patients he sends them to the out-patients' department of the Infirmary.

Mr. Sydney Holland : It is an old cry of the doctors, but our inquiries have inclined to show that such a state of things does not exist.

Sir William Cobbett : The out-patient is not admitted as a matter of course. Inquiries are made, and I have never heard any complaints from the doctors.

Mr. Lloyd George : Then they must all have come to me. (Laughter.) The doctors are more suspicious of the hospitals than they were of me, and that is saying a good deal. (Laughter.)

### Mr. Lupton's Views.

Mr. Charles Lupton, Leeds General Infirmary, said that one of the least expensive parts of a hospital from the lay manager's point of view was the out-patients' department. It really mattered very little whether they closed the out-patients' department or kept it on. They kept it on because it was undoubtedly affording a great benefit to people who would not otherwise receive satisfactory treatment. From the point of view of expense, it was a comparatively trivial matter. They had about forty-eight thousand new out-patients, of whom one-third might be affected by the Bill. The other two-thirds would still come to the Infirmary as they had done heretofore. Undoubtedly a section of the doctors felt that they were injured by the prevalence of hospitals in their midst, their objections being chiefly directed to the out-patients' department. Having had experience of nearly thirty years in the management of the Leeds Infirmary, and having been for ten years its chairman, he could speak with some experience upon the whole question. He doubted himself whether there was any solid reason for the doctors raising objection to the work done in the hospitals. There were certain persons who were jealous whatever happened, but a large section of the doctors worked hand in glove with the hospitals and endeavoured to work with them in every way they could. At Leeds they were endeavouring, by the aid of almoners, to prevent trivial cases from coming to them and cases which ought not to come to the hospitals at all. The fact was that there were very few people who had sufficient money to pay for their treatment who were prepared to wait for two or three hours in the out-patients' rooms before they could get attendance. They really did not see by what means they could keep going in the same way as in the past unless the State was prepared to pay them through the Insurance Bill for the actual work they did for people who were insured under the Bill.

### The German Fallacy.

Mr. Lloyd George : The income of the hospitals in Germany has gone up enormously since the introduction of their Insurance Bill.

Mr. Sydney Holland : There are no voluntary hospitals in Germany.

Mr. Lloyd George : But there are voluntary subscriptions.

Mr. Sydney Holland : For special purposes.

Mr. Lloyd George : I don't think it matters, from your point of view, what happens, so long as you get your income. The result of a scheme of national health like this is that people take an organised interest in health and won't see the hospitals go down.

Sir William Cobbett : The Lancashire working man's opinion would be that the fourpence covers the lot.

Mr. Lupton concluded his speech by saying that the workpeople subscribed very handsomely to the hospital fund. In Leeds they got over £7,000 a year from that source.

### The London Hospital.

Mr. Sydney Holland, representing the London Hospital, said that hospitals would be wanted just as much, if not all the more, under the new Bill. It was absolutely essential that the hospitals should not be hurt at all, because they were the only places where any progress was made in medicine and the only places where poor people could be treated for a very serious illness. That Bill was all very well for the people with illnesses which are not serious.

Mr. Lloyd George : Don't you consider pneumonia serious?

Mr. Holland : Pneumonia is essentially a case for skilled nursing.

Mr. Lloyd George : But you have not hospitals in every village. The Bill also deals with consumption, which is a fairly serious illness, with all respect to you. We are dealing with all serious diseases in cases where there are no hospitals. Where I was brought up we had no hospital for twenty miles. If we had had to depend on hospitals we should all have been dead.

Mr. Holland : All I can say is that serious cases are still left to the hospitals.

Mr. Lloyd George : I have read of cases even in London where the doctors are only called in to certify the cause of death.

Mr. Holland : I maintain that the hospitals must continue to deal with all serious cases and must not be disturbed. I suggest that in the Bill you might allow the hospitals to receive some payment for doing for the nation what it was paying the doctors to do. Why should not the hospitals receive some money for doing a great work for the nation?

Mr. Holland mentioned that the insurance of the nurses and employés at the London Hospital would cost them

£850 a year. They did not want to be insured, for they were all treated free when ill and provided with free medical attendance.

Mr. Lloyd George: It is true you treat them while they are at the hospital, but sickness comes on late in life, and what guarantee have you got that they will be properly treated when they are fifty or sixty years of age?

Mr. Holland: When nurses have left the hospitals they would not, I believe, come within the Bill because they are working independently, and are not employed persons.

Mr. Lloyd George: I thought they were generally employed by a nursing association.

Mr. Holland: They are on its books, but not employed by it.

Mr. Lloyd George: But the Bill provides for them as voluntary contributors. They can go on unless they are earning more than £160 a year, which few of them do. The nurse's life is a very hard one, and I am told that a great many of them break down early. If they break down hopelessly at thirty-five or forty, there is nothing but the hospital for them to fall back upon.

Mr. Holland: It is rather hard on us who have a very struggling existence to make us pay for people and give them benefits at the same time.

Mr. Lloyd George: Sir Wm. Cobbett's suggestion is that we ought to give you the credit for the work you do while the nurses are at the hospitals. That is a very good suggestion which I shall certainly consider.

Sir Savile Crossley, speaking on behalf of the Hospital Saturday Fund, said that the Fund was thirty-seven years old and had 350,000 subscribers. The Fund was strongly in favour of the maintenance of the voluntary system. The work done by that fund and similar bodies should be made known to the approved societies and the Health Committees, and if they could in any way help the Health Committees they would be very glad to do it.

Mr. Lloyd George, replying to the deputation, said that he was very glad to have had that interview with those competent to speak on behalf of the hospitals of the country and who commanded the confidence of the hospitals. He thought they were labouring under apprehensions which they would find would not be justified by the results. They feared that as a result of the Act the subscriptions would be considerably diminished. He did not think that there was reasonable ground to apprehend that consequence. There were many hospitals which depended upon substantial working-class contributions, but in future the working-class would put less in as far as members of Friendly Societies were concerned than they had hitherto paid towards the Friendly Societies. The Bill would relieve them of anything between sixpence and a shilling a month. He did not believe that men would neglect their duty towards the hospitals because they were calling upon them to make contributions towards sickness amongst their employés. He did not believe that that would be so. He did not think that that was compatible at all with what they knew of human nature. There might be a momentary irritation, but that appeared whenever they produced a Bill which called upon people to pay money. He was told three years ago that no more money would come to the hospitals. That happened with every Bill ever introduced which made people pay. People said that there was an end of all hospitals and charities; but human nature was much better than it liked to represent itself. After a momentary agitation had passed people would do their duty to those institutions in the same sort of way as hitherto. Those great institutions had been worked up through years and generations of labour, toil, sacrifice, and of anxiety, and they were naturally apprehensive that something would destroy the great work to which they had devoted their lives. He was not at all complaining of that. It was natural anxiety; but he did not think they would be justified. Even supposing they found it were so, that the hospitals were impoverished, that the sources of subscription dried up. The hospitals of the country were essentially a part of the machinery of civilisation, and he could not imagine any country allowing a single hospital to be closed. He did not want to indicate at the present moment what would have to be done, but he was perfectly clear in his own mind that no country could possibly allow a hospital to be closed because it was short of cash. If he were to indicate at the present moment, that would certainly be a more effective way of stopping subscriptions than anything in the Bill. No Government could allow a single hospital to be closed. In Germany the hospitals had developed, grown, and strengthened since their Insurance Bill was brought in, because it educated public opinion as to the need for hospitals and as to the important part it played in healing, and that was more important for hospitals than almost anything which they could imagine in order to induce people to subscribe. Sir William Cobbett had suggested that there should be some contribution from the insurance fund to the work which hospitals would be rendering towards insured persons. They had given power in the Bill to approved societies to contribute. The deputation would probably say that that was not sufficient; but the remedy was very largely in their own hands. Mr. Sydney Holland had said that two of the classes who used the hospital were discontented patients who were not satisfied with their doctors and the discontented doctors who sent their patients. They should say to the first class of persons, "Are you insured?" If they were, then they had no right to come to the hospitals. They should further tell the doctors that they couldn't take their burden off their shoulders. Mr. Holland complained that they had not legislated for hospitals. It was for the very reason that they believed in the voluntary system and wanted it to continue. If they wanted to be put upon a fund they must be put upon it under the same conditions as any other institutions, and there would have to be a certain amount of Government control over their work. They were in a position to say to the people, "We cannot take you unless you are prepared to meet us. We are free contracting parties."

Mr. Holland: We cannot refuse a person who is desperately ill simply because some one declines to pay for him.

Sir William Cobbett: Hospitals cannot refuse patients.

Mr. Lloyd George: You cannot refuse the individual, but you can in future be in a position to insist on help from the approved societies when they get contributions from the State and enforced contributions from employers.

Mr. Lloyd George continued that in regard to Clause 12 the suggestion had been made that in certain cases the sick pay might in one form or other be made a contribution to the hospital itself. That was worth considering, and it would undoubtedly very greatly improve the financial position of the hospital. He was prepared to meet them in regard to the suggestion that where a hospital undertook to pay the wages of a nurse during the time that she was ill and to undertake the medical attendance upon her, then the Government would be prepared to reduce the charge by the amount of the sick pay. That, he thought, would be a reduction of about twopence a week, but he was getting actuarial advice. That would apply to the case of all employés where there was a firm contract. There was no reason why they should not get whatever sum of money was set aside for medical attendance, but the question of medical attendance was one for them to discuss with the doctors. He had had as much trouble with the doctors as he really wanted. They were more jealous of the hospitals than they were of the Chancellor of the Exchequer. If they could come to an arrangement with medical men, it would be much easier for him afterwards. Directly he came to an arrangement in one particular section, he offended another and got no support from the former, who by that time had found a fresh grievance. It was of very little use to him unless they came to him having arranged matters with the medical profession, and they were far from having done that at the present moment. It was a difficult business. They were really trying to do their best for the national health. They had raised enormous sums of money for the purpose. Everybody agreed that it must be done, but the moment they attempted to do it everybody had their own individual grievance and raised difficulties. He was not complaining of that, but he would be glad to find out where that fifteen millions of money was supposed to be going. He had not discovered anybody yet who was receiving it. Everybody said they were worse off than before, but that could not be the case. He would be very much obliged if it were possible for them to talk the matter over with the medical profession, and then come to him with some sort of arrangement with regard to cases which came to the hospitals and were taken off the hands of the doctors. He wished them to remember that it was not true that they were dealing only with trivial cases. He would consider very carefully all the suggestions they had made, and he hoped that they would be able to meet them in some of the points.

Mr. A. Wm. West having thanked Mr. Lloyd George, the deputation withdrew.

